# A Case of Traumatic Proptosis

**DOI:** 10.1155/2013/514328

**Published:** 2013-03-07

**Authors:** Bobby Desai

**Affiliations:** Department of Emergency Medicine, University of Florida College of Medicine, 1329 SW 16th Street, P.O. Box 100186, Gainesville, FL 32610-0186, USA

## Abstract

We present a case of traumatic proptosis in a competitive football player. This entity can occur with a significant decrease in vision, but in this case it did not. Some other causes of this condition are also discussed. A review of some traumatic conditions that may cause proptosis is provided as well.

## 1. Case

A 23-year-old previously healthy male was playing football and was tackled during practice. During the course of the tackle, his helmet dislodged and struck in his right eye. Per the patient, his eye was examined by the team physician and he was cleared to resume practice. When the patient awoke the next day, he blew his nose, and felt that his right eye “popped out.” After the event, he felt that there was pressure behind his eye. His mother presented with the patient to the Emergency Department and showed the physicians a photo from the prior day and it was obvious that the eye seemed more protuberant on the day of arrival to the ED. The patient complained that his vision is blurry at baseline, no change today. The patient denied loss of consciousness at the time of incident. He complained of moderate pain and blurred vision with no foreign body sensation.

On arrival, his vital signs were temperature 36.7 degrees Celsius, pulse 67 beats per minute, respiratory rate 20 breaths per minute, and blood pressure 143/88 mmHg. His visual acuity was 20/20 on the left and 20/80 on the right. His review of systems was positive for only eye pain and blurred vision. Except for his eye exam, his physical exam was unremarkable. The left eye exam was normal. His eye exam revealed the right eye protuberant compared to the left eye, a subconjunctival hemorrhage on the right, normal pupillary shape and the pupils were reactive and not sluggish. There was no overt evidence of globe rupture.

**Figure 1:**
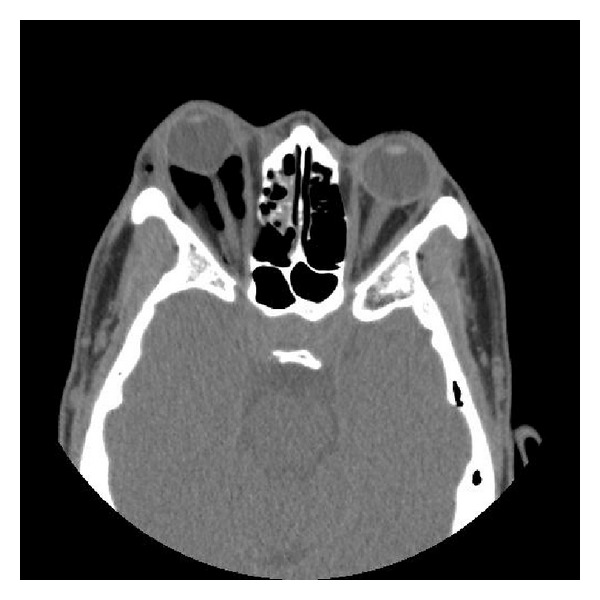
Diffuse intraconal/preseptal air and severe proptosis of the right eye resulting in tenting of the optic globe and stretching of the optic nerve.

**Figure 2:**
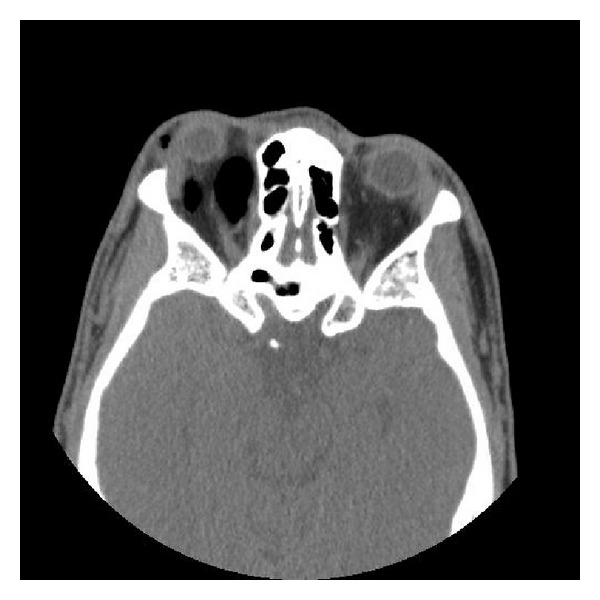
Diffuse intraconal/preseptal air and severe proptosis of the right eye resulting in tenting of the optic globe and stretching of the optic nerve.

**Figure 3:**
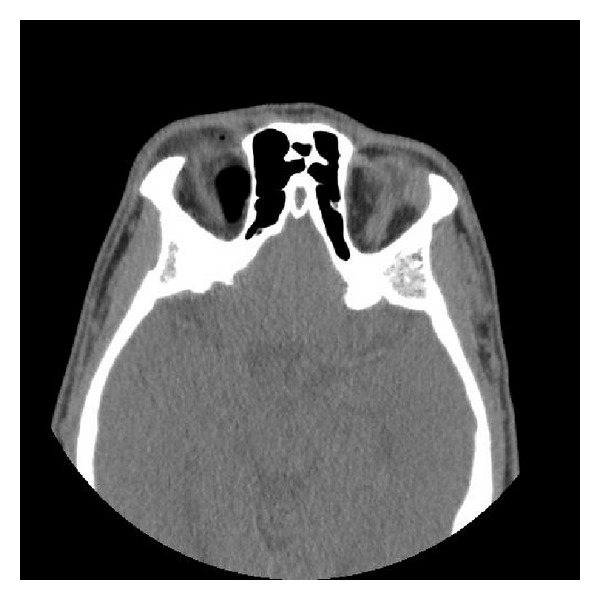
Diffuse intraconal/preseptal air and severe proptosis of the right eye resulting in tenting of the optic globe and stretching of the optic nerve.

The patient was sent for a computed tomography (CT) scan of his brain and orbits. The CT scan revealed diffuse intraconal/preseptal air with severe proptosis of the right eye. This resulted in tenting of the optic globe with stretching of the optic nerve. There was air (retrobulbar emphysema) noted on the scan, which was thought to originate from a fracture of the right lamina papyracea. There was also a small Tenon space hematoma on the right side. There was no evidence of entrapment of the extraocular muscles (Figures 1, 2, and 3).

**Figure 4:**
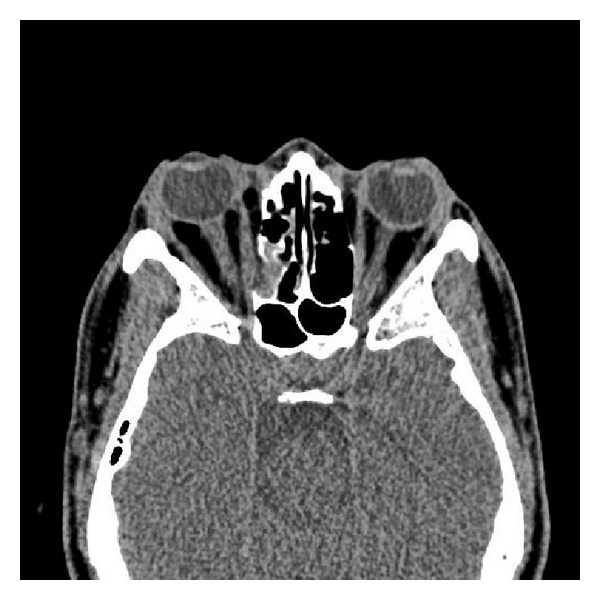
Medial orbital wall blowout injury with relief of tension orbit. It also shows more than half the gas has been resorbed and no definite evidence of optic sheath injury.

During the course of his stay in the ED, the patient complained of worsening vision and was provided pain medication with some relief. However, approximately two hours later, the patient again began to complain of pain. Ophthalmology was at the bedside and placed gentle pressure on the globe. The patient felt a decrease in his pain and was sent back to CT scan for a postreduction view of his eye (Figures 3 and 4). A protective shield was placed over his eye and his was subsequently discharged home and was followed up one week later with no vision abnormalities.

## 2. Discussion

Proptosis may be caused by a number of etiologies, medical and surgical, traumatic and nontraumatic, and can occur in anyone from soldiers to housewives [1, 2]. Furthermore, proptosis can have more than one process concurrent. Some of these include orbital hemorrhage and orbital emphysema [2, 3].

Orbital hematomas can be caused by trauma and can be classified as intraorbital or subperiosteal. Intraorbital hematomas are more common and show findings of subconjunctival hemorrhage, lid edema and bruising and diminished ocular movement. Subperiosteal hematomas, which occur secondary to rupture of subperiosteal blood vessels, are not as common and will present with proptosis, lid ecchymosis, and impairment of eye movement [4]. Nontraumatic hemorrhages can occur secondary to increases in intraocular pressures such as forceful vomiting and diving. Depending on location, optic nerve function can be compromised [5–7]. The management of the hematoma depends on how impaired vision is; without evidence of visual disturbance, the hemorrhage can be observed without specific treatment. When vision is affected, the hematoma should be evacuated in consultation with an ophthalmologist. The diagnosis of orbital or retro-orbital hematoma is typically made via imaging, usually with computed tomography which can also detect the presence of orbital wall fractures.

Other traumatic events can cause proptosis, including orbital wall fractures; 50% of orbital wall fractures have orbital emphysema. The medial orbital wall and floor of the orbit connect with sinus spaces that if injured, could directly lead into air entering the orbital spaced from the sinus [3, 8]. Orbital emphysema can also occur without fractures and can occur spontaneously as well. Usually, air within the orbit causes no issues except for transient proptosis and diplopia unless there is a ball-valve mechanism preventing air escape, in which case optic nerve or retinal ischemia can take place due to air tamponading the vessels supplying the retina and optic nerve [9]. There are various techniques including lateral canthotomy and needle aspiration to decompress the orbit if visual compromise is present. 

Subluxation of the globe may be defined as an anterior displacement of the globe distal to the orbital rim and eyelid retraction posteriorly. This may occur due to both traumatic and nontraumatic causes. Acute subluxation results in corneal exposure and the potential for abrasions and ulceration due to lack of protection from the eyelid and lack of moisture which can potentially affect vision. Vision may also be affected due to the stretching of the optic nerve. This can be treated with gentle repositioning of the globe back into the orbit [10]. Use of lubricating drops, ointments, or even steroids may be prescribed in consultation with an ophthalmologist.

Traumatic avulsion of the eye is uncommon and may cause damage to the optic nerve and sheath separately or in tandem. Extraocular muscles may be complete dehisced or remain attached to the globe. Complete avulsion with loss of vision requires enucleation and prosthetic replacement.
